# Lymph but Not Blood Vessel Invasion Is Independent Prognostic in Lung Cancer Patients Treated by VATS-Lobectomy and Might Represent a Future Upstaging Factor for Early Stages

**DOI:** 10.3390/cancers14081893

**Published:** 2022-04-08

**Authors:** Melanie Biesinger, Nele Eicken, Alexander Varga, Michael Weber, Milos Brndiar, Georg Erd, Peter Errhalt, Klaus Hackner, Sarah Hintermair, Alexander Petter-Puchner, Axel Scheed, Elisabeth Stubenberger, Bahil Ghanim

**Affiliations:** 1 Department of General and Thoracic Surgery, Karl Landsteiner University of Health Sciences, University Hospital Krems, 3500 Krems an der Donau, Austria; melanie.biesinger@krems.lknoe.at (M.B.); n.eicken@hotmail.com (N.E.); a.varga@ordination-pathologie.at (A.V.); milos.brndiar@krems.lknoe.at (M.B.); georg.erd@krems.lknoe.at (G.E.); sarah.hintermair@krems.lknoe.at (S.H.); alexander.petter-puchner@krems.lknoe.at (A.P.-P.); axel.scheed@krems.lknoe.at (A.S.); elisabeth.stubenberger@krems.lknoe.at (E.S.); 2 Statistics Consultancy, Karl Landsteiner University of Health Sciences, 3500 Krems an der Donau, Austria; michael.weber@kl.at; 3 Department of Pneumology, Karl Landsteiner University of Health Sciences, University Hospital Krems, 3500 Krems an der Donau, Austria; peter.errhalt@krems.lknoe.at (P.E.); klaus.hackner@krems.lknoe.at (K.H.)

**Keywords:** lung cancer, staging, lymph vessel invasion, blood vessel invasion, prognostic factor

## Abstract

**Simple Summary:**

Lung cancer is the second most common cancer entity and the leading cause of cancer-related deaths worldwide despite constant advantages in immune-oncology and personalized medicine. Invasion of the lymphatic and vascular system has been investigated as a risk factor in the past and available data suggested a strong prognostic impact. However, many studies did not distinguish between the invasion of the lymphatic and the vascular system. Furthermore, this issue has not yet been studied in a homogenous cohort treated by a VATS lobectomy and staged by the most recent 8th edition of the TNM staging. We hence examined the role of lymphatic and vascular invasion as two separate risk factors in NSCLC patients treated by a modern minimal-invasive VATS lobectomy to re-encourage the scientific interest in the prognostic role of vascular invasion and to further improve NSCLC risk stratification.

**Abstract:**

Lung cancer is the most frequent cause of cancer-related death worldwide. The patient’s outcome depends on tumor size, lymph node involvement and metastatic spread at the time of diagnosis. The prognostic value of lymph and blood vessel invasion, however, is still insufficiently investigated. We retrospectively examined the invasion of lymph vessels and blood vessels separately as two possible prognostic factors in 160 patients who underwent a video-assisted thoracoscopic lobectomy for non-small-cell lung cancer at our institution between 2014 and 2019. Lymph vessel invasion was significantly associated with the UICC stage, lymph node involvement, tumor dedifferentiation, blood vessel invasion and recurrence. Blood vessel invasion tended to be negative prognostic, but missed the level of significance (*p* = 0.108). Lymph vessel invasion, on the other hand, proved to be a prognostic factor for both histological subtypes, adenocarcinoma (*p* < 0.001) as well as squamous cell carcinoma (*p* = 0.018). After multivariate analysis apart from the UICC stage, only lymph vessel invasion remained independently prognostic (*p* = 0.018). Remarkably, we found analogue survival curve progressions of patients with stage I, with lymph vessel invasion, compared to stage II non-small-cell lung cancer. After further validation in prospective studies, lymph vessel invasion might be considered as an upstaging factor in resectable lung cancer. Especially in the early-stage of the disease, it might represent an additional risk factor to consider adjuvant therapy after surgical resection.

## 1. Introduction

Non-small-cell lung cancer (NSCLC) is the second most common malignancy worldwide and the leading cause of cancer-related death, translating to an annual death roll of 1.8 million patients [1]. Survival probability and clinical treatment allocation are distinctly related to the patient’s tumor stage [2,3].

The TNM staging system was developed over 70 years ago by Pierre Denoix in France, resulting in the Union for International Cancer Control (UICC) TNM staging. Denoix already categorized the tumor according to its tumor size and invasiveness in the T-stage, according to its lymph node invasion in the N-stage, and according to its distant metastases in the M-stage [4,5]. The TNM staging for lung cancer has—similar to other malignancies—changed regularly over recent decades, resulting in its most recent 8th edition published in 2017 [6]. Surprisingly, given the immense progress in the understanding of tumor biology resulting in modern personalized medicine, the TNM staging criteria for lung cancer basically remained the same since the first classification.

In the past, a series of studies investigated the prognostic impact of lymphovascular invasion, comprising the invasion of lymph and blood vessels, on the outcome of lung cancer patients. The promising initial data suggested a correlation of lymphovascular invasion with a shorter cancer-specific and overall survival [7]. Nevertheless, up to now, lymphovascular invasion has not been taken into consideration, neither in the latest staging, nor in the treatment recommendations [6,8].

In contrast to the tumor’s local lymphatic and blood vessel invasiveness, the tumor size and lymph node invasion are decisive for treatment allocation. A survival benefit for adjuvant chemotherapy was shown for patients with lymph node metastases [9]. The current guidelines hence recommend adjuvant chemotherapy after primary resection in early-stage NSCLC in any case of lymph node metastases or a tumor size over 4 cm [6,8]. For patients with the N0 situation, however, data are still contrary. In stage IA, a survival disadvantage was associated with the use of adjuvant chemotherapy [9] and accordingly, no adjuvant chemotherapy is recommended for tumors up to 3 cm without lymph node metastases [6]. Contrary to stage IA, stage IB (tumor size 3–4 cm, N0) patients might benefit from adjuvant therapy, however, the current data are insufficient and guidelines regarding stage IB are not precise [6,9,10].

As mentioned above, not only the lymph node state seems to have a prognostic impact, but also the lymphovascular invasion. However, most studies investigating this topic applied the meanwhile outdated 7th edition of the TNM staging, or even older classifications, and several focused on early-stage patients only [7]. Furthermore, most previous studies did not pathologically differentiate between the invasion of lymph and blood vessels [11,12,13,14,15,16,17,18,19,20,21,22]. Only few studies investigated lymph and blood vessel invasion separately and, according to these scarce data, lymph vessel invasion seems to be the stronger prognostic factor.

Accordingly, we re-evaluated specifically LVI and BVI as prognostic factors in NSCLC, more precisely, in patients receiving minimal-invasive modern video-assisted-thoracoscopy (VATS)-lobectomy. We hypothesized LVI and BVI being prognostic when analyzed within the recent 8th edition of the TNM classification.

Based on the here-presented retrospective study, we want to reopen the discussion of whether LVI should be considered as a precursor of lymph node invasion and if this factor should be taken into account for treatment decisions—especially in stage I NSCLC without lymph node metastases but with LVI. We hypothesize that the “L status” should be regarded as an upstaging factor in early-stage NSCLC and should be validated by further studies.

## 2. Materials and Methods

### 2.1. Patients

The retrospective single center study was conducted after obtaining ethical approval of the local ethics committee of the Karl Landsteiner University for Health Sciences (1055/2019). The study was performed in accordance with the Declaration of Helsinki. The statistical analysis was based on retrospectively collected pseudonymized data.

**Figure 1:**
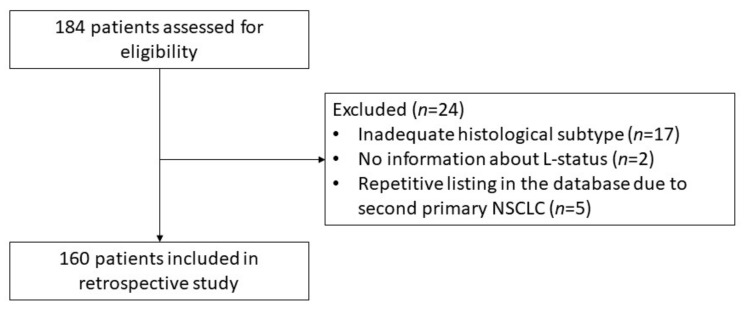
Consort diagram visualizing the exclusion of patients. Abbreviations: L, Lymphatic Invasion; NSCLC, Non-Small Cell Lung Cancer.

One hundred and eighty-four consecutive patients with NSCLC who underwent VATS-lobectomy between 2014 and 2019 at the Department of General and Thoracic Surgery, University Hospital Krems, were identified for possible enrollment. Patients were excluded in case of inadequate histological subtyping in the pathological report, or in case of large cell adenosquamous or neuroendocrine tumors. In addition, in some cases, the patient appeared twice in the database because of a second primary NSCLC, or the histopathological report gave no information about the L-status. Excluding those patients resulted in 160 patients eligible for inclusion in the study (Figure 1).

After the diagnosis of NSCLC, all patients were presented in a multidisciplinary team to determine the treatment plan. Preoperative evaluation included staging examinations as well as cardiac and pulmonary assessment according to the current ESMO guidelines [6,23].

Patients were generally staged with CT scan and PET-CT. If the mediastinal lymph nodes were radiologically suspicious, staging was—according to the ESMO/ESTS guidelines—initially supplemented by EBUS and, if further necessary, by mediastinoscopy [6].

Generally, patients were admitted at least one day prior to surgery for preoperative work-up. If further diagnostic steps were necessary, they we admitted 2–3 days prior to surgery.

Following oncological guidelines, with every VATS-lobectomy, a systematic mediastinal lymphadenectomy was performed as en bloc resection of lymph nodes and the surrounding mediastinal fat. At least three mediastinal lymph node levels were resected, always including the subcarinal lymph nodes [6,24].

### 2.2. Pathological Analysis

The resected pulmonary lobe, as well as all resected lymph nodes, were analyzed pathologically to define the postoperative TNM staging. All patients were classified according to the 8th edition of TNM staging and those treated between 2014–2017 were re-categorized according to the formerly documented tumor size, lymph node status and metastases. Six (3.8%) patients were re-allocated to stage IIA in the 8th edition instead of IB in 7th edition with tumor sizes of 4–4.9 cm and, hence, undertreated in the past in accordance with the then-eligible guidelines.

The invasion of lymphatic and blood vessels was evaluated using histomorphological evaluation alone or with additional immunohistochemical evaluation.

There is no difficulty in histomorphological differentiation between arteries and lymphatic vessels because of the characteristic anatomical structure, including layers of smooth muscle in arterial blood vessels. In contrast, the differentiation between lymphatic vessels and small venous blood vessels can be difficult as they are often lacking a visible layer of smooth muscle and have a similar microscopical structure. To differentiate between the invasion of lymphatic vessels and blood vessels’ immunohistochemistry can be performed. Podoplanin, a transmembrane mucoprotein, is expressed by lymphatic but not blood vessel endothelium, therefore, it can identify the lymphatic nature of a vessel. In contrast, CD34, a transmembrane sialomucin glycoprotein, shows expression in all endothelial cells, either lymphatic or blood vessels [7,25,26].

The invasion of lymphatic vessels and blood vessels was indicated as L-status and V-status, respectively.

### 2.3. Statistical Analysis

For all statistical analyses, IBM SPSS Statistics 26 was used. In all analyses, *p*-values below 0.05 were considered significant and only two-sided *p*-values were used for the reported analyses. The applied statistical analyses are listed in the figure and table legend.

Cancer-free survival (CFS) was defined as time between day of surgery and date of diagnosis of recurrence or last follow-up. The pathologically verified LVI is indicated as L-status (L1, L0), blood vessel invasion as V-status (V1, V0), the letter N was used for lymph node metastases, and M for distant metastases.

Local recurrence was defined as recurrence within the ipsilateral chest cavity of the primary tumor, whereas distant recurrence was defined as contralateral thoracic or extrathoracic recurrence.

Pearson’s chi^2^, or Fisher’s exact test were performed to compare groups of categorial data. Kaplan–Meier plot was applied for the estimation of survival, and the Log-rank test to calculate the *p*-values. For multivariate survival calculations, the Cox proportional hazard analysis was chosen, thereby examining the prognostic impact of the clinicopathological variables’ UICC stage, histology type, grading, lymphatic invasion, blood vessel infiltration and adjuvant therapy.

## 3. Results

In summary, 160 patients (104 males, 56 females, mean age 65 ± 8.6, range 26–86 years) who underwent a VATS lobectomy between 2014 and 2019 for NSCLC were included in the study. The median postoperative hospital stay was eight days (range of 4–29 days).

The rather long hospital stay was partly due to postoperative complications, such as postoperative arrhythmias which made cardiological treatment and work-up necessary, or a prolonged air leak which resulted in a postponement of discharge. Furthermore, it was also partly due to our enhanced recovery-after-surgery program which includes intense support and mobilization. 

The 30-day mortality was 0% and the median follow-up-time was 27 months (range of 0–68).

One hundred and seventeen patients (73.1%) were alive and recurrence-free at the last follow-up, whereas 35 (21.9%) were diagnosed with cancer recurrence and 29 patients (18.1%) died. Some patients who died had a recurrence first, resulting in an overlap.

Macroscopic and microscopic tumor-free resection margins were achieved for all patients, resulting in a 100% R0 rate. The median number of resected lymph nodes was 13 (range of 5–44). The overall survival rate after surgery for all patients was 91% for one year, 81% for three years and 67% for five years.

Most of the cases were pathologically classified as early-stage disease, resulting in 100 (62.5%) patients presenting with postoperative UICC stage I, whereas 33 (20.6%) were classified as stage II, 23 (14.4%) as stage III and four (2.5%) as stage IV NSCLC. Of the latter, three were initially staged as oligometastatic M1b (two patients with single cerebral metastases and one with unilateral adrenal metastases). After downstaging by successful cerebral stereotactic radiation or radio-chemotherapy, respectively, all these patients were re-evaluated by the multidisciplinary tumor-board and found eligible for curative surgery.

One patient had to be up-staged after surgery to stage M1b due to an incidental finding of a small pleural nodule during the surgery, which was later diagnosed as pleural dissemination in the final pathological result.

All patients were re-evaluated in the multidisciplinary tumor-board after the final pathological staging and 64 patients (40%) in stages IB to IV were allocated to adjuvant treatment whereas 96 (60%) received surveillance only. 

Overall, more than one third of all patients (*n* = 59, 36.9%) presented with LVI and thus were classified as L1. LVI was strongly associated with the UICC staging. Only 18 (18.0%) of the 100 stage I cases were diagnosed with LVI. With 18 out of 33 patients, more than half of the stage II patients already showed LVI (54.5%). Of 23 patients in the UICC stage III, 20 (87.0%) were classified as L1, whereas three out of four (75.0%) stage IV patients proved to suffer from L1-tumors. Expectedly, the presence of LVI was significantly associated with the presence of lymph node metastases (*p* < 0.001) as LVI can also be interpreted as a precursor of lymph node involvement. All patients with an N1-status also had an L1-status. Interestingly, two patients with mediastinal lymph node metastases (N2) showed no LVI. Of all patients without any lymph node metastases, in 22 cases LVI was observed, as opposed to 99 patients with an N0–L0-status. Additionally, the presence of LVI correlated significantly with a higher degree of tumor dedifferentiation (*p* < 0.001) and blood vessel infiltration (BVI, *p* < 0.001). Of 24 patients with detected BVI, 23 (95.8%) also showed LVI (Table 1). These results support the hypothesis that LVI translates to higher tumor aggressiveness and invasiveness.

Regarding the histological subtype, the proportion of L1 patients did not differ significantly between adenocarcinoma (ADC) and squamous cell carcinoma (SQCC) of the lung.

The presence of lymphatic invasion was significantly correlated with the occurrence of postoperative recurrence (*p* < 0.001, Table 1), whereas BVI missed statistical significance (*p* = 0.158). The influence of LVI on the site of recurrence is given in Table 1. LVI was a risk factor for both local as well as distant recurrence, and only 30% of all L1 patients were recurrence-free.

The key clinicopathological characteristics of the total study population, L0- and L1-patient cohorts, are demonstrated in Table 1.

The chi-square test showed no significant association between the resected lobe and the L-status. However, this might be explained by the relative low numbers in the corresponding subgroups. Concerning lung function parameters, only FEV1, but not DLCO, showed a tendency towards lower values in the L-positive group. A larger sample size might reveal significant results. Furthermore, logistic regression did not show a significant relationship of the combined FEV1, DLCO, and the resected lobe and the L-status. Therefore, these parameters did not influence the presence of lymphatic invasion. In contrast to these findings, the logistic regression showed—in line with the chi square results—a significant relationship between the V- and the L-status (*p* < 0.001).

The patients’ characteristics were also analyzed with regard to the V-status. The parameters, i.e., grading, the UICC, N-status, and distant metastasis, showed significant differences between the V1 and the V0 group. As expected, the L-status also showed significant differences between the V1 and V0 group, as did, vice versa, the V-status between the L1 and L0 group. However, as the V-status has no significant influence in the survival analysis (see below) we focused, in the following, on the L-status as the prognostic parameter and provided the detailed overview of the patients’ characteristics in Table A1 in Appendix A.

### 3.1. Univariate Survival Analyses

**Figure 2:**
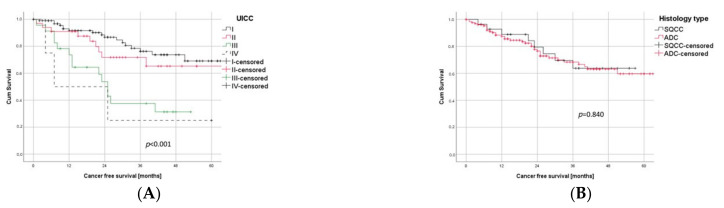

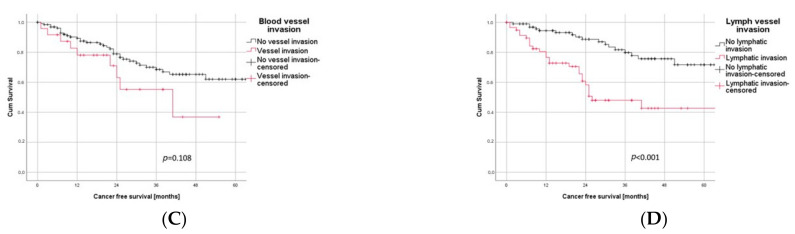
Prognostic Factors in Univariate Survival Analysis. (**A**) Survival differences between the four UICC stages (log rank test: *p* < 0.001). Stage III and IV were combined, both separately being under-represented due to the inclusion criteria of minimally invasive surgery. (**B**) Histological subtype did not significantly correlate with survival (log rank test: *p* = 0.840). (**C**) BVI showed a tendency to being negative prognostic but missed level of significance (log rank test: *p* = 0.108). (**D**) LVI was significantly (log rank test: *p* < 0.001) associated with worse outcome.

As to be expected, CFS significantly correlated with the UICC stage (*p* < 0.001, Figure 2A), whereas the histological subtype had no impact on survival (*p* = 0.84, Figure 2B). The presence of BVI showed a tendency towards a worse outcome in univariate analyses, but with a *p*-value of 0.108, it did not reach the level of significance (Figure 2C). 

LVI, on the other hand, was significantly (*p* < 0.001) associated with a negative prognosis in univariate analysis. The impact of LVI on the postoperative CFS is visualized in Figure 2D, with a 1-year, 3-year, and 5-year CFS of 94% vs. 81%, 80% vs. 48%, and 72% vs. 43% for L0 vs. L1, respectively. 

**Figure 3:**
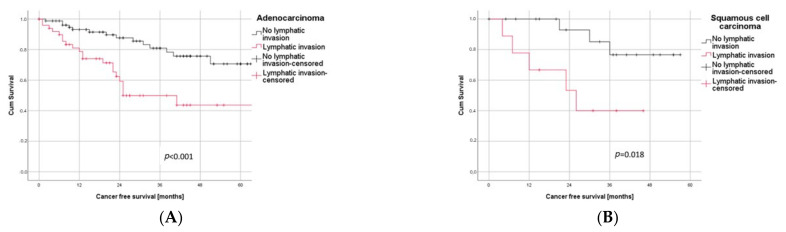
Subgroup Analysis. (**A**) Patients with adenocarcinoma and LVI had a significantly worse outcome than those without LVI (log rank test: *p* < 0.001). (**B**) In patients with squamous cell carcinoma, LVI significantly correlated with negative outcome (log rank test: *p* = 0.018).

In a subgroup analysis, LVI was significantly associated with a poor outcome for both histological subgroups, ADC (*p* < 0.001) as well as SQCC (*p* = 0.018), as illustrated in Figure 3A,B.

**Figure 4:**
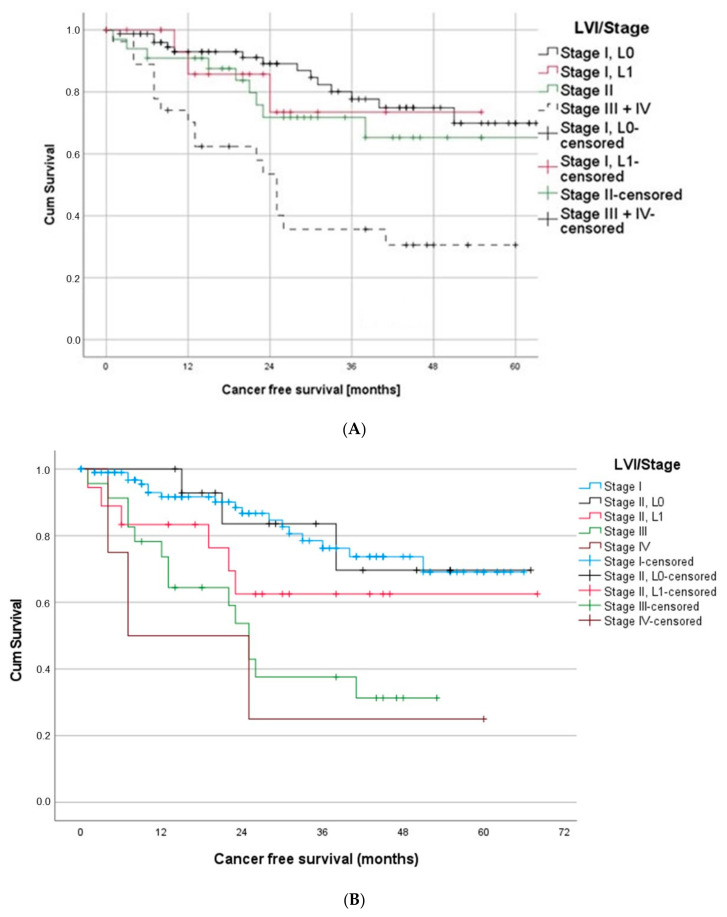

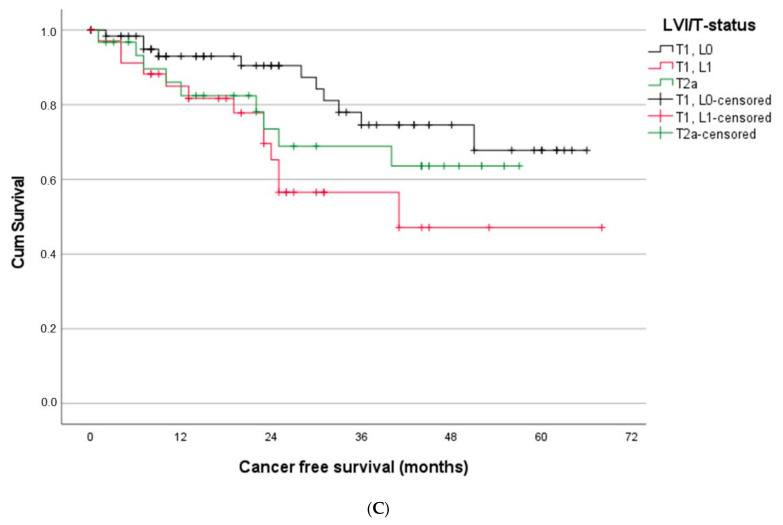
(**A**) Patients suffering from stage I/L1 had a similar outcome as stage II patients. (**B**) Patients with stage II/L1 and stage III showed a similar outcome, whereas stage II L0 patients had significantly better outcome than stage III patients. (**C**) Stage I patients with T1L1 showed significantly worse prognosis compared to T1L0 patients and, in the long run, also compared to patients with T2a.

To further analyze the impact of LVI on the outcome of the early stages of the disease, we then focused on the UICC stage I cases. Most interestingly, the survival curve of stage I/L1 is very similar to the curve of stage II (Figure 4A). In contrast, a stage I, L0 patient showed the most promising survival with 93%, 78% and 70% CFS after one, three and five years.

Analogous results were found when comparing survival curves of stage II and stage III. The survival curves of stage II L0 patients differed noticeable from stage III. However, stage II L1 patients and stage III patients showed similar progressions (Figure 4B).

To analyze stage I patients in more detail, we also looked specifically at patients with a T1N0 tumor. Most interestingly, the survival of T1L1 patients was significantly worse compared to T1L0 (log rank test: *p* = 0.014). Moreover, the survival curve of T2a was initially similar to the one of T1L1 and even better after 22 months. These first results suggest a similar survival impact of the L-status, as already observed in the past for the invasion of the visceral pleura [27].

### 3.2. Multivariate Survival Analyses

After showing that LVI was a prognostic factor in univariate analyses, we next aimed to evaluate its independence from the major clinical characteristics’ UICC stage, histology, grading, BVI and adjuvant chemotherapy through multivariate analysis. Apart from the UICC stage, only LVI was independently associated with CFS, translating to a hazard ratio (HR) of 3.139 (95% confidence interval (CI) 1.217–8.093) (Table 2). These results indicate that after radical minimal invasive lung cancer resection, LVI is a strong independent negative prognostic factor.

## 4. Discussion

In the current study, we analyzed the prognostic impact of LVI and BVI as two separate prognosticators in NSCLC patients. We aimed for a homogenous study cohort, including only patients receiving a modern VATS lobectomy with a systematic mediastinal lymphadenectomy. To our knowledge, these are the first data regarding the prognostic value of LVI and BVI restricted to patients eligible for minimal-invasive thoracic surgery. In contrary to most other authors [11,12,13,14,15,16,17,18,19,20,21,22], we analyzed the impact of lymphatic and blood vessel invasion separately in multivariate survival analyses and applied the 8th edition of TNM staging.

Due to the inclusion criteria of thoracoscopic surgery, we included mainly patients diagnosed at an early-stage of the disease. However, according to the most recent ESMO guidelines, also patients with an oligometastatic disease and stage III patients are represented in our real-life database [28]. Our data confirmed an independent prognostic value of LVI for patients undergoing a VATS lobectomy. A subgroup analysis confirmed the prognostic impact of LVI for both histological subtypes, adenocarcinoma and squamous cell carcinoma. The presence of LVI correlated significantly not only with the UICC stage, but also with occurrence of lymph node metastases, poor tumor differentiation, postoperative recurrence and with blood vessel infiltration, but not with the histological subtype. These results are in line with the hypothesis that LVI is to be seen as a sign of an aggressive and more invasive tumor biology in both adenocarcinoma as well as squamous cell carcinoma of the lung.

BVI, in contrast, showed a tendency towards a negative prognostic impact, but narrowly missed statistical significance in univariate analysis. However, BVI might turn out to be of prognostic value when evaluated in a larger sample size, e.g., within big registries.

Additional analysis of the prognostic impact of LVI on the different stages of the disease showed similar survival curve progressions of patients with a stage I and L1 status compared to stage II NSCLC and, analogously, a similar survival curve of stage II L1 patients and stage III patients.

By analyzing the T-stage in more detail, again, we found an even worse prognosis for T1a patients with LVI compared to T1a L0, but also, compared to T2a. Taken together, these results show that on the level of the UICC stage, but also on the level of the T-stage, patients with LVI must expect a survival comparable with the next higher UICC stage. This leads us to the conclusion that the detection of LVI should be regarded as an up-staging factor. Therefore, intensified treatment decisions should be considered. According to our data, patients with stage I L1 and T1a L1 might benefit from adjuvant chemotherapy and the beneficial effect should be investigated in larger studies and within a prospective study design. In multivariate survival analysis, only the UICC stage and LVI remained prognostic, thus confirming that LVI is an independent prognostic parameter in resectable lung cancer.

Two decades ago, the so-called lymphovascular invasion had already become the subject of research as a promising prognostic factor in NSCLC [7], but it has not yet found consideration in current guidelines. As mentioned before, numerous studies reported a prognostic impact, especially for stage I [11,12,13,15,16,17,18,19,20,22,29,30,31,32,33,34,35]. However, most studies included tumor sizes up to 5 cm as UICC stage I, according to the 6th or 7th edition [35,36,37]. Tumor sizes of 4 to 5 cm, however, have been upstaged in the meantime to stage IIA, within the most recent 8th edition, and historic and present data regarding the impact of lymphatic invasion for stage I are, therefore, inconsistent [38].

Overall, the data situation is quite inhomogeneous. Some studies included only adenocarcinoma histology [12,15,19,39], some compared squamous cell carcinoma and adenocarcinoma and found analogous results for both subtypes [11,13,16,17,18,22,30,31,40], whereas others found a prognostic value of LVI only for adenocarcinoma [14,32,41]. Several study cohorts were heterogenous regarding the extent of surgery, including patients who underwent a lobectomy, but also patients with sublobar resection or a pneumonectomy [11,13,14,16,17,18,19,22,29,30,31,32,33,39,40,42]. Additionally, most authors either did not report or not perform a systematic mediastinal lymphadenectomy in all patients [13,15,18,19,29,30,31,33,35,40,42]. However, both of these two factors—the extent of resection and lymphadenectomy—are well known to affect survival significantly [24]. In addition, the surgical approach, thoracoscopic versus open surgery, was heterogenous within the study cohorts or was not reported at all [11,13,14,16,18,22,30,32,33,40]. We, therefore, only included patients after a VATS lobectomy with a systematic lymphadenectomy, since this is oncologically required and relevant for reproducible survival data.

In our opinion, the main drawback of many former studies, however, is the fact that most researchers did not differentiate between the infiltration of lymph or blood vessels [11,12,13,14,15,16,17,18,19,20,21,22] and in some studies the terms lympho- and angiovascular invasion were not specified by the authors at all [33,34,39,42,43].

The commonly used terms lymphovascular or angiolymphatic invasion are both generally used as a collective term for lymph vessel invasion and intratumoral blood vessel infiltration [32]. Additionally, the pathological differentiation for blood and lymph vessel invasion might be challenging and immunostaining is not always available or applied for pathological examination, as already criticized by Mollberg and colleagues [7].

Consequently, the different effects of LVI and BVI are not yet clear and both factors should thus be examined separately. Without an exact definition and reproducibility of a prognostic factor, its prognostic value and evaluation in clinical practice is hardly practical.

In the here-presented study, we differentiated between these two factors—primarily regarding their prognostic value on the one hand, but also regarding their impact on the site of recurrences on the other hand. Uni- and multivariate analysis revealed only LVI as a negative prognostic factor in contrast to BVI. Nevertheless, we suggest an evaluation of the prognostic value of both parameters in larger cohorts.

Concerning the site of recurrence, preliminary data are controversial—some authors reported a correlation of the vascular invasion of lymph and blood vessels with early local recurrence [31], others with distant metastasis [11,18,21]. Kato and colleagues did differentiate between lymphatic invasion and intratumoral vascular invasion and found both to be independent negative prognostic factors for adenocarcinoma, but not for non-adenocarcinoma. However, no significant correlation between the site of recurrence and the kind of infiltrated vessel was reported [32]. A recent study of Lee et al., on the contrary, focused exclusively on BVI and found a significant correlation with systemic, but not with locoregional, recurrence [44].

In our study, LVI but not BVI was associated with recurrence. LVI was identified as a risk factor for both local and distant recurrence, however, this was not the case for BVI. Thus, four patients with BVI and distant metastases are opposed to 13 patients without BVI suffering from distant metastases (data not shown). This indicates that in our patient cohort, LVI was the superior marker for predicting CFS, which is also reflected in the survival analyses.

Some authors already proposed lymphovascular invasion as a possible upstaging factor in NSCLC especially in early-stages, without differentiating between lymph and blood vessel invasion. Ruffini et al., for example, reported similar survival curves of stage IA patients with lymphovascular invasion and stage IB patients without invasion [14], whereas Tsuchiya et al. found an even worse prognosis of stage IA patients with lymphovascular invasion compared to IB patients without invasion [18]. Both, however, applied the 7th edition, categorizing tumor sizes up to 5 cm as IB.

Our data showed comparable results, but selectively for LVI with a survival curve of T1, with LVI (corresponding stage IA) resembling the survival curve of T2a (corresponding to stage IB)—classified according to the current 8th edition of TNM staging. In line with these findings, we found that, for stage I and stage II patients with LVI, survival curves correspond with the next higher stage.

We therefore specify the suggestion and consider the invasion of lymph vessels as a stronger and independent negative prognostic factor also within the 8th edition of the TNM staging system.

The most recent guidelines recommend adjuvant therapy for stage II lung cancer. For stage IA, on the other hand, no adjuvant therapy is currently recommended, and for stage IB (tumor sizes from 3–4 cm and no lymph node involvement) adjuvant chemotherapy is only recommended in case of additional risk factors [6].

We want to re-open the discussion of whether a positive L-status in stage I NSCLC should count as such a risk factor, as it appears to be a precursor of lymph node metastases, as also reflected by the strong association between the N- and the L-status in our study cohort. 

In view of our results, we suggest positive LVI as an up-staging factor that might result in the recommendation of adjuvant chemotherapy after curative surgery in stage I with lymph vessel involvement.

Preliminary data already proved a positive effect of adjuvant chemotherapy for stage I patients with vascular invasion [18,45]. However, both study cohorts were heterogenous regarding the extent of parenchyma resection and lymph node resection, as well as the kind of infiltrated vessel.

Of note, the prognostic impact of lymphovascular invasion and the effect of adjuvant therapy are currently also under investigation for various extrathoracic cancer types. A worse prognosis, association with lymph node involvement and higher recurrence rates for patients with lymphovascular invasion have been shown, amongst others, for different gastrointestinal and urogenital malignancies, and for melanoma and thyroid cancer [46,47,48,49,50,51,52,53,54]. Some studies also proved a survival benefit of adjuvant chemotherapy or radiation in case of lymphovascular invasion [48,51,53].

For NSCLC, we hope that our results re-encourage the discussion of the prognostic impact of LVI, especially for early-stage NSCLC with regard to its potential relevance for treatment decisions.

Study Limitations: As a major limitation of our study, we see the retrospective study design, the single-center nature, as well as the limited case number of 160 patients. However, this reflects the fact that we defined our inclusion criteria strictly in regard to the extent of resection and the surgical approach, leading to less eligible patients. The sample size might explain the non-significant *p*-value in some calculations, e.g., FEV1 vs. L-status.

It would be interesting to evaluate if, in larger study cohorts—contrary to our initial results—blood vessel infiltration also proves to be prognostic.

As a further limitation of our work, we see the relatively short follow-up period, with a median follow-up of 27 months, which is mainly due to the inclusion of patients resected between 2014 and 2019, resulting, also, in the inclusion of patients with surgery less than two years ago.

Furthermore, it is clear that the retrospective study design might lead to the inhomogeneous distribution of important clinical prognosticators within the analyzed groups (L0 vs. L1). In addition, the exclusion of the confounders influencing the prognostic value of the L-status by propensity score-matching was not efficient since the L-status had to be regarded as a precursor of the N-status and, accordingly, also as a precursor of the late stage of the disease. Thus, we relied on multivariate analyses only, to investigate if the prognostic value of the L-status is influenced by other clinical and pathological characteristics.

However, our first results indicate a strong and independent prognostic power of LVI after minimal invasive lung cancer surgery compared to BVI and we recommend verification of these results in larger surveys.

## 5. Conclusions

The invasion of lymph vessels but not blood vessels is an independent negative prognostic factor for NSCLC patients undergoing a VATS-lobectomy. Preliminary data for stage I patients suggest a prognosis of stage I with LVI comparable to the prognosis of stage II patients, and furthermore, a similar survival of stage II patients with LVI compared to stage III patients.

Further studies investigating the prognostic impact of LVI in more homogenous and prospective study cohorts are preferable. Furthermore, sufficient data regarding the prognostic benefit of adjuvant therapy, especially for patients with a negative lymph node status but with LVI, are mandatory to evaluate the necessity of adapting the treatment recommendations for stage I NSCLC.

## Figures and Tables

**Table 1:** Association of lymph vessel invasion with the patients’ clinicopathological parameters (*n* = 160).

Characteristic		Total *n* = 160 (%)	L0 *n* = 101 (%)	L1 *n* = 59 (%)	*p*-Value
Mean age in years (±SD)		65 ± 8.6	65 ± 9.3	66 ± 7.4	0.41 #
Gender	female	56 (35)	40 (71.4)	16 (28.6)	0.11 *
	male	104 (65)	61 (58.7)	43 (41.3)	
Comorbidities	No diabetes	136 (85)	89 (65.4)	47 (34.6)	0.148 *
	Diabetes	24 (15)	12 (50)	12 (50)	
	No hypertension	78 (48.8)	55 (70.5)	23 (29.5)	0.059 *
	Hypertension	82 (51.2)	46 (56.1)	36 (43.9)	
	No COPD	104 (65)	68 (65.4)	36 (34.6)	0.419 *
	COPD	56 (35)	33 (58.9)	23 (41.1)	
	No CHD	137 (85.6)	88 (64.2)	49 (35.8)	0.478 *
	CHD	23 (14.4)	13 (56.5)	10 (43.5)	
Resected lobe	RLL	38 (23.8)	28 (73.7)	10 (26.3)	0.181 *
	RUL	51 (31.9)	29 (56)	22 (43.1)	
	LLL	27 (16.9)	18 (66.7)	9 (33.3)	
	LUL	35 (21.9)	22 (62.9)	13 (37.1)	
	RML	7 (4.4)	2 (28.6)	5 (71.4)	
	RUL + RML	2 (1.3)	2 (100)	0	
Smoking	Never smoker	24 (15.3)	17 (70.8)	7 (29.2)	0.429 *
	Smoker	133 (84.7)	83 (62.4)	50 (37.6)	
Lung function ^1^	FEV1 ≥ 80%	96 (61.1)	65 (67.7)	31 (32.3)	0.189 *
	FEV1 < 80%	61 (38.9)	35 (57.4)	26 (42.6)	
	DLCO ≥ 80%	70 (49)	43 (61.4)	27 (38.6)	0.715 *
	DLCO < 80%	73 (51)	47 (64.4)	26 (35.6)	
Histology	SQCC	29 (18.1)	20 (69)	9 (31)	0.471 *
	ADC	131 (81.9)	81 (61.8)	50 (38.2)	
	Grading well or moderately differentiated ^2^	92 (58.6)	68 (73.9)	24 (26.1)	<0.001 *
	Grading poorly differentiated ^2^	65 (41.4)	30 (46.2)	35 (53.8)	
Staging	N0	121 (75.6)	99 (81.8)	22 (18.2)	<0.001 *
	N1	21 (13.1)	0	21 (100)	
	N2	18 (11.3)	2 (11.1)	16 (88.9)	
	UICC I	100 (62.5)	82 (82)	18 (18)	<0.001 *
	UICC II	33 (20.6)	15 (45.5)	18 (54.5)	
	UICC III	23 (14.4)	3 (13)	20 (87)	
	UICC IV	4 (2.5)	1 (25)	3 (75)	
	M0	155 (97.5)	99 (63.9)	56 (36.1)	0.117 *
	M1a	1 (0.6)	0	1 (100)	
	M1b	3 (1.9)	1 (33.3)	2 (66.7)	
	V0 ^2^	134 (84.8)	98 (73.1)	36 (26.9)	<0.001 *
	V1 ^2^	24 (15.2)	1 (4.2)	23 (95.8)	
Recurrence	No recurrence	124 (78)	87 (70.2)	37 (29.8)	<0.001 *
	Local	13 (8.2)	7 (53.8)	6 (46.2)	
	Distant	17 (10.7)	6 (35.3)	11 (64.7)	
	Local and distant	5 (3.1)	0	5 (100)	

^1^ In 3 cases, pulmonary function testing was performed extramural and the written result was not available in the patients’ chart retrospectively at the time of data collection. In further 14 cases, the DLCO was not measured and data are consequently missing. ^2^ The pathological result did not describe grading in 3 cases and V-status in 2 cases, respectively, and data were hence not available retrospectively. Abbreviations: L1, Lymph vessel invasion; L0, No lymph vessel invasion; COPD, Chronic obstructive pulmonary disease; CHD, Coronary heart disease; RLL, Right lower lobe; RUL, Right upper lobe; LLL, Left lower lobe; LUL, Left upper lobe; RML, Right middle lobe; FEV1, Forced expiratory volume in 1 s; DLCO, Diffusing capacity for carbon monoxide of the lung; SQCC, Squamous cell carcinoma; ADC, Adenocarcinoma; N, Lymph node metastases; UICC, Union of International Cancer Control; M, Presence of metastases; V1, Blood vessel invasion; V0, No blood vessel invasion. # *t*-test. * Chi square test.

**Table 2:** Lymphatic vessel invasion correlated significantly with shorter cancer-free-survival.

Multivariate Analysis			
Characteristic	Significance	HR	95% CI
UICC I	0.051	0.233	0.054–1.004
UICC II	0.029	0.204	0.049–0.851
UICC III	0.390	0.560	0.150–2.098
UICC IV	0.056	1	
Histology type	0.882	1.063	0.476–2.375
Grading	0.471	1.277	0.657–2.482
LVI	0.018	3.139	1.217–8.093
BVI	0.168	1.868	0.768–4.543
Adjuvant therapy	0.976	0.983	0.337–2.867

HR, Hazard ratio; CI, Confidence interval; UICC, Union of International Cancer Control; LVI, lymphatic vessel invasion; BVI, blood vessel invasion.

## Data Availability

Upon reasonable request, all data and material are available from the corresponding authors.

## Appendix A

**Table A1:** Association of blood vessel invasion with the patients’ clinicopathological parameters (*n* = 158).

Characteristic		Total *n* = 158 ^1^ (%)	V0 *n* = 134 (85%)	V1 *n* = 24 (15%)	*p*-Value
Gender	female	54 (34)	46 (85)	8 (15)	*p* = 0.925 *
	male	104 (66)	88 (85)	16 (15)	
Comorbidities	No diabetes	134 (85)	113 (84)	21 (16)	*p* = 0.690 *
	Diabetes	24 (15)	21 (88)	3 (13)	
	No hypertension	77 (49)	68 (88)	9 (12)	*p* = 0.232 *
	Hypertension	81 (51)	66 (81)	15 (19)	
	No COPD	102 (65)	85 (83)	17 (17)	*p* = 0.485 *
	COPD	56 (35)	49 (88)	7 (13)	
	No CHD	135 (85)	115 (85)	20 (15)	*p* = 0.750 *
	CHD	23 (15)	19 (83)	4 (17)	
Resected lobe	RLL	37 (23)	33 (89)	4 (11)	0.694 *
	RUL	51 (32)	41 (80)	10 (20)	
	LLL	26 (16)	22 (85)	4 (15)	
	LUL	35 (22)	31 (89)	4 (11)	
	RML	7 (4)	5 (71)	2 (29)	
	RUL + RML	2 (1)	2 (100)	0 (0)	
Smoking	Never smoker	22 (14)	19 (86)	3 (14)	0.864 *
	Smoker	133 (86)	20 (15)	113 (85)	
Lung function ^1^	FEV1 ≥ 80%	94 (61)	83 (88)	11 (12)	0.173 *
	FEV1 < 80%	61(39)	49 (80)	12 (20)	
	DLCO ≥ 80%	68 (48)	56 (82)	12 (18)	0.679 *
	DLCO < 80%	73 (52)	62 (85)	11 (15)	
Histology	SQCC	28 (18)	25 (89)	3 (11)	0.467 *
	ADC	130 (82)	109 (84)	21 (16)	
	Grading well or moderately differentiated ^1^	91 (59)	83 (91)	8 (9)	0.006 *
	Grading poorly differentiated ^1^	64 (41)	48 (75)	16 (25)	
Staging	N0	120 (76)	107 (89)	13 (11)	0.016 *
	N1	21 (13)	16 (76)	5 (24)	
	N2	17 (11)	11 (65)	6 (35)	
	UICC I	99 (63)	89 (90)	10 (10)	0.015 *
	UICC II	33 (21)	28 (85)	5 (15)	
	UICC III	22 (14)	15 (68)	7 (32)	
	UICC IV	4 (3)	2 (50)	2 (50)	
	M0	154 (97)	132 (86)	22 (14)	0.040 *
	M1a	1 (1)	0 (0)	1 (100)	
	M1b	3 (2)	2 (67)	1 (33)	
	L0	99 (63)	98 (99)	1 (1)	<0.001 *
	L1	59 (37)	36 (61)	23 (39)	
Recurrence	No recurrence	122 (78)	106 (87)	16 (13)	0.296 *
	Local	13 (8)	11 (85)	2 (15)	
	Distant	17 (11)	13 (76)	4 (24)	
	Local and distant	5 (3)	3 (60)	2 (40)	

^1^ The pathological result did not describe grading in 3 cases and V-status in 2 cases, respectively, and data were, hence, not availably retrospectively, resulting in 158 patients with V-status. ^2^ In 3 of the total 160 cases, pulmonary function testing was performed extramurally and the written result was not available in the patients’ chart retrospectively at the time of data collection. In further 14 cases, the DLCO was not measured and data are consequently missing. Abbreviations: L1, Lymph vessel invasion; L0, No lymph vessel invasion; COPD, Chronic obstructive pulmonary disease; CHD, Coronary heart disease; RLL, Right lower lobe; RUL, Right upper lobe; LLL, Left lower lobe; LUL, Left upper lobe; RML, Right middle lobe; FEV1, Forced expiratory volume in 1 s; DLCO, Diffusing capacity for carbon monoxide of the lung; SQCC, Squamous cell carcinoma; ADC, Adenocarcinoma; N, Lymph node metastases; UICC, Union of International Cancer Control; M, Presence of metastases; V1, Blood vessel invasion; V0, No blood vessel invasion. * Chi square test.
